# Novel porcine repetitive elements

**DOI:** 10.1186/1471-2164-7-304

**Published:** 2006-12-01

**Authors:** Ralph T Wiedmann, Dan J Nonneman, John W Keele

**Affiliations:** 1 USDA, ARS U. S. Meat Animal Research Center, P. O. Box 166, Clay Center, NE, USA

## Abstract

**Background:**

Repetitive elements comprise ~45% of mammalian genomes and are increasingly known to impact genomic function by contributing to the genomic architecture, by direct regulation of gene expression and by affecting genomic size, diversity and evolution. The ubiquity and increasingly understood importance of repetitive elements contribute to the need to identify and annotate them. We set out to identify previously uncharacterized repetitive DNA in the porcine genome. Once found, we characterized the prevalence of these repeats in other mammals.

**Results:**

We discovered 27 repetitive elements in 220 BACs covering 1% of the porcine genome (Comparative Vertebrate Sequencing Initiative; CVSI). These repeats varied in length from 55 to 1059 nucleotides. To estimate copy numbers, we went to an independent source of data, the BAC-end sequences (Wellcome Trust Sanger Institute), covering approximately 15% of the porcine genome. Copy numbers in BAC-ends were less than one hundred for 6 repeat elements, between 100 and 1000 for 16 and between 1,000 and 10,000 for 5. Several of the repeat elements were found in the bovine genome and we have identified two with orthologous sites, indicating that these elements were present in their common ancestor. None of the repeat elements were found in primate, rodent or dog genomes. We were unable to identify any of the replication machinery common to active transposable elements in these newly identified repeats.

**Conclusion:**

The presence of both orthologous and non-orthologous sites indicates that some sites existed prior to speciation and some were generated later. The identification of low to moderate copy number repetitive DNA that is specific to artiodactyls will be critical in the assembly of livestock genomes and studies of comparative genomics.

## Background

Repetitive elements comprise ~45% [1] of mammalian genomes and are increasingly known to impact genomic function by contributing to the genomic architecture, by direct regulation of gene expression [2,3] and by affecting genomic size, diversity and evolution [4-8]. The ubiquity and increasingly understood importance of repetitive elements (REs) contribute to the need to identify and annotate REs [9]. In recent years, several attempts have been made to automate the process of *de novo *identification and characterization of REs [10-16]. The algorithms take into account the likely evolutionary history of the REs – not only genetic drift, but also the processes that lead to the juxtaposition of REs [10]. Because knowing the evolutionary history of each RE helps to define the type of RE, these algorithms are valuable not only in identifying repetitive sequence, but also in increasing our understanding of the evolutionary role of the identified RE. Our initial attempt was to identify novel repetitive DNA with a program called RECON [10], which produced 14,067 families of REs with 249 of those having count numbers of 10 or more. We decided a different approach was needed that would organize closely related elements in a parsimonious way. In this paper, we describe 27 novel porcine repetitive elements and estimate their prevalence in swine and other species.

## Results

We identified repetitive elements using a procedure similar to previously published methods [10,11]. First, we used RepeatMasker [17] on the BAC sequences to mask out previously characterized repeat elements. Second, we identified all pair-wise alignments among masked sequences using BLAST [18]. Third, we identified multiple copy sequence segments with alignments to many sites (≥ 10). Fourth, we clustered sites linked by pair-wise alignments and constructed phylogenetic trees. Fifth, excessive variation (2-fold) in copy number within a putative RE caused it to be divided; co-localization of RE among many sites caused them to be merged. Sixth, we examined flanking sequences of putative RE for clues about replication machinery or to consolidate RE that should be merged. Seventh, we estimated the prevalence of RE in an independent set of porcine sequences as well as in the genomes of other species.

### Our method compared to RECON

The bulk of the automated parts of our process, Steps 2 through 4, were very similar to RECON [4]. RECON does not appear to have analogues for Steps 1 (RepeatMasker), 5, 6, or 7. We utilized Step 1 to steer us away from previously characterized repetitive. We utilized the manually intensive Steps 5–7 to achieve a more parsimonious (smaller number of repeat families) than appeared to be possible with RECON alone. In this sense, we envision that our method is a complement to RECON, not a replacement.

### Steps 1 – 4

Thirty-six percent of the sequence was masked by RepeatMasker. Comparing all unmasked sequence fragments (≥ 50 bp) produced 1,334,953 pair-wise alignments. One thousand five hundred seventy-nine highly redundant sequences (totaling 1.07 Mb) were identified that had a minimum of 10 hits for at least 50 contiguous bases. Sixty putative repeat element families resulted from clustering the 1579 highly redundant sequences. The repeat element families were labeled MPRE1 – MPRE60 (for Meat Animal Research Center Porcine Repetitive Element). Their lengths ranged from 55 to 1059 bp and their copy numbers (across the 220 discovery BACs) ranged from 12 to 1102.

### Steps 5 – 6

The 60 original MPREs were consolidated into 31 because of overlap or co-localization at multiple sites. Twenty-nine MPREs were absorbed into 31; the 31 original MPRE identifiers of the longer sequences were kept to maintain provenance. In addition, there were three combinations (MPRE20 and 57; MPRE15, 17, 19 and 26; MPRE44, 50 and 52) of repeats that frequently appeared together in the same order with some variation in their relative spacing. The most consistent group contained two elements – MPRE20 in reverse complement followed by a small gap, then MPRE57. All thirteen times that MPRE20 occurred, it occurred in this grouping. MPRE57 occurred 13 out of 14 times in this grouping. Naturally, we concluded that MPRE20 (600 bp) and MPRE57 (204 bp) were two parts of a longer RE that had a variable middle (100–250 bp range for all but one example). After examining the alignment in ClustalX, we could see that the middle was conserved except for an 84 bp deletion in one instance and a 67 bp insertion in another. Further review of the BACs showed that the 13 groups containing MPRE20 and MPRE57 sometimes occurred in overlapping regions between pairs of clones in the BAC collection, meaning that we only had 7 unique loci plus one very unique locus that had a PRE1a (Porcine Repeat Element 1a, as identified by RepeatMasker) inserted into the gap. There was no pattern to the gap in the other instances. We include this longer repeat element in our list of novel porcine repeat elements as MPRE61, which is more fully described in a later section.

The final alteration to the list of MPREs was the removal of MPRE48 due to its low copy number in the set of 275,595 porcine BAC-ends supplied by the Wellcome Trust Sanger Institute (hereafter shortened to "Sanger") [19]. Surprisingly, MPRE48 was found to appear less frequently, only six times, in BAC-ends (335.9 Mb) than in the much smaller portion of the genome spanned by the set of fully sequenced BACs (36.4 Mb) from which the MPREs were derived. That brings the final number of novel repeat elements reported here to 27, although we decided against removing MPRE48 from the fasta file of MPREs, see Additional file 1.

### Step 7

Table 1 lists the MPREs along with their observed count numbers in the TIGR (The Institute of Genomic Research, Rockville, MD) *Sus scrofa *Gene Index [20] and the Sanger BAC-end sequences [19]. Noting that the data set of BAC-ends is 4.8 times larger than the TIGR Gene Index (104,328 entries of expressed swine sequence totalling 70.0 Mb), we conclude that all the novel repeats occur less frequently in expressed sequence than in genomic DNA.

The prevalence of these newly identified REs was compared to that of known REs. Three of the newly discovered porcine REs, MPRE11, 16 and 38, were more common than the LINE element L3 and one, MPRE42, was about as common as L3 (Table 1). The other 23 MPREs have lower count numbers. In the Sanger archive of 275,595 BAC-ends, the number of elements for all SINEs was 203,206, for all LINES was 116,107 and for all LTRs (Long Terminal Repeats) was 25,066 based on RepeatMasker. Looking specifically at the LINEs, the most common by far was L1 with 94,325, followed by L2 with 18,720 and L3 is third with 2,358.

These newly discovered repeat elements did not appear to be duplicated genes, LINE elements or expressed sequence that was transposed by a LINE element. To address these questions, the MPREs were translated and compared (BLAST) to the GenBank nr database and only one strong hit was found. MPRE1 hit *Sus scrofa *interferon alpha-1 precursor with a bit score of 352, so it was eliminated from further consideration as a novel RE. For comparison, the highest bit score of MPREs reported here was less than 50. The repeats were also compared (BLAST) to vectors, mitochondrial DNA, and tRNAs. The middle of MPRE58 did have high similarity to tRNA-GLU; otherwise, there were no substantial high-scoring pairs.

## Discussion

Certain difficulties arise when defining repeat elements. One is that REs often are present as mosaics of smaller subsets of commonly occurring sequences [21,22]. Another is that REs can often sustain considerable mutations, including large truncations and insertions. Two extreme examples of this are the truncation of the 5' end during retrotransposition, and the insertion of one RE into the middle of another. A third difficulty requiring resolution is that segmental duplication will create very long repeated sequences that do not retro-transpose together, and therefore should be broken up into their retro-transposable component parts. RECON, the software for identification of REs described by Bao and Eddy, handles all three of these difficulties [10].

Our approach was intentionally a bit more simplistic. We were able to create a much more parsimonious set of RE than what we were able to generate with RECON. Whereas RECON intends to recreate the full repeat elements in the way that will make for the best possible additions to the RepeatMasker database, as well as aid in the study of the evolutionary history of the repeat elements, our goal was to mask out the most commonly repeated regions of the porcine genome. The technique we found most useful in refining the definitions of the MPREs was to plot the frequency of BLAST hits as a function of position within the sequence of the putative repeat elements. From the criteria used to define them, the number of hits was at least 10 across the whole sequence – but many showed a much higher hit frequency along part of their lengths. For purposes of comparison, we applied RECON to our pair-wise alignments from Step 2. RECON divided the 1,334,953 BLAST hits into 29,631 potential repeat elements that were then grouped into 14,067 families. Only 249 of these families had 10 or more elements. Note that it is possible for a family containing only one element to correspond to many BLAST hits. Rather than continue with so many families, we found that our method yielded a more parsimonious classification of moderately repetitive elements. One difference between the two methods was that our method required a minimum copy number prior to the formation of families of repeat elements.

The MPREs have no clear connections to known proteins. The NCBI BLASTX results for these sequences were typically a combination of description-less accessions and unrelated proteins in a variety of organisms. That remained true when the dataset was compared to the TIGR gene index for *Sus scrofa *[20].

The novel repeat elements were compared to known types of repeats – SINEs, LINEs and LTRs – and did not fit the definitions for those classes of repeat elements. Because RepeatMasker would mask out low-complexity regions, the methods used here would not initially find the tail ends of LTRs. Each MPRE was tested for nearby low-complexity regions and none were consistently found. One of the characteristics of SINEs is the presence of tRNA coding sequence in their 5 prime regions [23,24]. Only MPRE58 had a region similar to tRNA, and that was in the middle of its sequence. LINEs are best characterized by their two ORFs – one coding for a reverse transcriptase and the other for a protein with RNA binding activity [6]. All the MPREs were translated to potential proteins and compared to a comprehensive database (NCBI BLASTX). None of the results were similar to the possible translations of a LINE.

### Counting repeat elements is challenging

Because of the degeneracy of repetitive elements it is difficult to arrive at an accurate count in the target genome. Another difficulty in the quantification of repeat elements is that REs are often composed of smaller repeat units that occur more frequently than the larger unit [21,22].

To characterize the prevalence of MPREs, we went to an independent data set, the Sanger BAC-ends from the CHORI-242 library archived at Ensembl [19]. Table 1 lists three different measures of prevalence of MPRE within these BAC-ends. The first measure (BLAST hits to BAC-ends) gives the number of hits that were at least half the length of the repeat element. An issue here is the typical size of the traces – an average of 1219 bp. The longer REs will tend to be under-counted due to edge effects in the trace archive. The next two measures of count number were calculated by plotting the number of BLAST hits as a function of position on the RE. Some of the resulting plots were smooth and flat across most of the RE with an expected drop-off near each end. For these "regular" plots the count number was the average value of the middle 90% of the plot amplitude. Other plots varied quite a bit in amplitude across the RE. This was likely due to sub-repeats that hit in areas of the genome that the whole repeat did not. During this measure of count number there was no lower limit to the size of the hit other than that needed to get the expectation value below 0.1. These were considered irregular and the algorithm for determining their count number was to take the smallest value on the plot after ignoring the first and last 10% of the plot. A few plots were only mildly irregular, and for those both the regular and irregular algorithms were used with both numbers reported in Table 1.

### Comparing the novel repeat element content across genomes

The sequences of novel porcine repetitive elements listed here were compared (BLAST [25]) to a recent build of the complete cow genome (AAFCO2 from [26]) as well as against the mouse and human genomes. In the case of mouse, there were no significant similarities found. The comparison to the human genome yielded only one significant hit – a 37 bp long section of MPRE17 (870 bp long) matched once in chromosome 9 thousands of bp away from any annotated features. The comparison to the cow genome yielded a variety of results. Five of the 27 MPREs did not hit at all (MPREs 6, 22, 28, 50 and 60), and three others (MPREs 44, 49 and 61) had ten or fewer hits (Table 1), despite the fact that the cow genome contains ten times more sequence than the collection of porcine BAC-ends tested. Fourteen of the 27 MPREs appeared frequently in cow as well as pig, as indicated by having at least 1000 BLAST hits to the cow genome.

**Figure 1:**
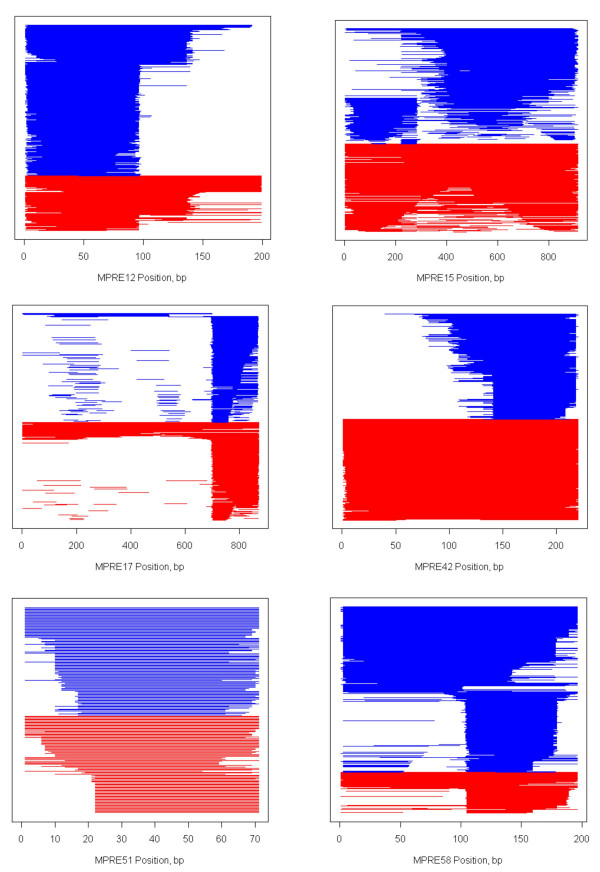
**Distribution of BLAST hits to cow and pig DNA across selected MPREs**. BLAST hits plotted across MPREs 12, 15, 17, 42, 51 and 58. Along the abscissa lies each MPRE sequence and stacked above are the corresponding hits to the cow genome in blue and to pig BAC-ends in red. The hits are ordered from the top down by length.

Not surprisingly, the bovine hits tend to be shorter than the porcine hits because the MPREs were defined from pig sequence and as such would be expected to be more intact in porcine. What is interesting is that in both species the endpoints of the hits have a strong tendency to line up to particular spots in the MPRE, as shown in Figure 1 using MPRE12, 15, 17, 41, 51 and 58 as examples. Sometimes the common endpoints are the same in both species, sometimes not. This could be a result of the repeat elements being comprised of smaller repeat elements, not all of which have the same frequency of occurrence in either genome. The longer MPREs often had more than one sub-region with multiple extra hits. This, too, could be evidence of internal repeat structure.

**Figure 2:**
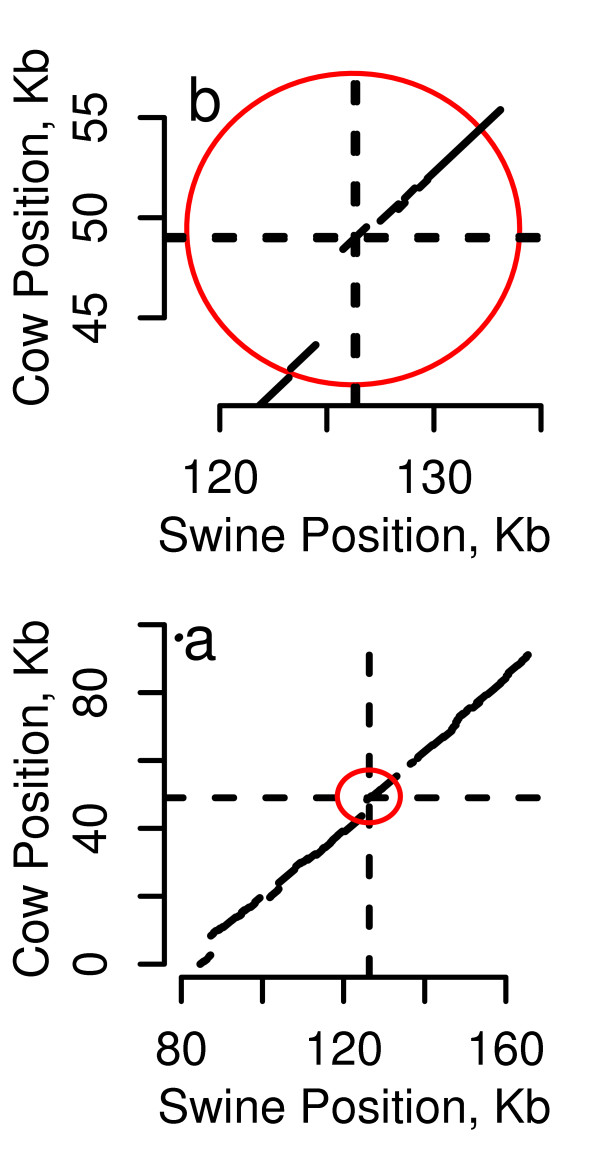
**(a and b) – MPRE55 in homologous positions in pig and cow**. MPRE55 exists in homologous positions in pig and cow. Along the horizontal axis lies the pig BAC with accession number AC147198. Along the vertical axis lies the cow BAC with accession number AC138165. The numerous line segments are BLAST hits between the two BACS that have bit scores of at least 100. Dashed lines are drawn through the positions on the BACs where MPRE55 is located. The circle indicates the region containing MPRE55 that is expanded and shown in **Figure 2(b)**.

Figure 2 shows that MPRE55 occurs in both swine and cattle in orthologous loci. The pig BAC lies along the x-axis, and the cow BAC lies along the y-axis. Also plotted are line segments of high similarity between the two BACs. The preponderance of these segments demonstrates little genomic rearrangement between species, which indicates that these are orthologous regions of likely common ancestry between the two species. This region is highly similar to the human contig NT_005403.16 and the locus of MPRE55 corresponds to the 3' UTR of the model gene LOC643405, which codes for a protein similar to TGF-beta induced apoptosis protein 2.

Because the collection of BACs spans only 1% of the whole pig or cow genome, we cannot rule out the possibility that all of the MPREs have at least one orthologous location in both species. The fact that 12 MPREs did not have blast hits in any of the cow BACs makes it seem likely that those 12 are relatively recent evolutionary occurrences. Of the 10 MPREs that appear most frequently in the cow, only two, MPRE55 and MPRE59, were observed to appear in orthologous locations among the tested set of fully-sequenced BACs.

**Figure 3:**
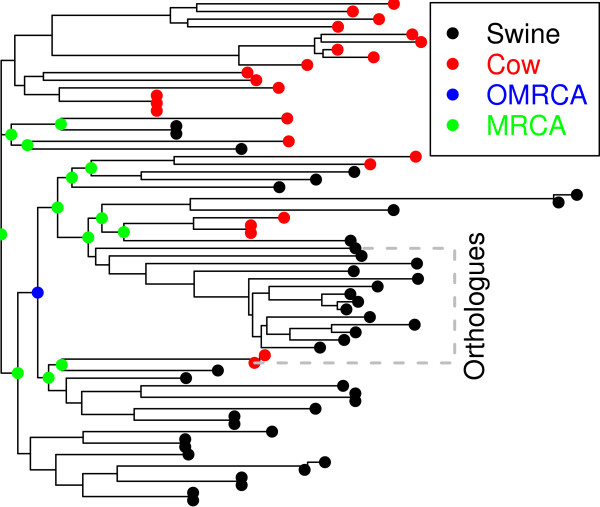
**Phylogeny of MPRE55 in pig and cow**. The phylogram displays the BLAST hits obtained from querying MPRE55 against the fully sequenced BAC libraries for pig and cow. The red dots indicate examples of MPRE55 from the cow and the black dots indicate pig examples. The orthologous sites depicted in Figure 2 are noted by the grey dashed lines and the word "orthologues." Also shown are the Most Recent Common Ancestors (MRCA) between species in green and, in blue, the MRCA for the 2 orthologues (OMRCA). In both cases the BACs covered about 1% of the total genome. The MRCA lie within a relatively narrow band of time consistent with a single speciation event and there appears to be considerable radiation among elements following speciation (i.e., time frame spanning MRCA).

A phylogenetic analysis was performed on the different integration sites of MPRE55 from both the cow and pig BAC libraries using ClustalX (see Additional file 2 for the sequences), and the output (Additional file 3) was then input into R [27] to create Figure 3. The sequences that occurred at orthologous locations in swine and cattle are highlighted. As expected, the pig branches and cow branches tend to be separate. It is notable that the most similar sequences that occur in both species do not come from orthologous locations, but seem to be found in loci that originated after the cow and pig ancestral lines diverged. The evolutionary distance between them is represented by the sum of horizontal distances that one must travel along the tree to connect the two sequences. The leftmost part of that path represents a common ancestor. It is not surprising that the two sequences in question have individually diverged a significant amount from the original sequence of the common ancestor at that locus. The more surprising result is that some of the pig and cow sequences are more similar to each other than the sequences at the oldest loci. Coincidental convergence is an unlikely possibility. A more likely explanation is that enough copies of the old sequence were created that some of them experienced much less mutation than the diverged sequences at the ancestral locus. The most recent common ancestors (MRCA) occurred in a narrow window of time (evolutionary) relative to the full extent of the tree (< 1/5 of the distance from the root to the most peripheral branch). The MRCA among the orthologous sites occurred within the same time frame as the other MRCA. The tree clearly shows considerable radiation following speciation as evidenced by large genetic distances from MRCA to peripheral tips.

### A closer look at MPRE61

Allelic differences or SNP can be identified from cases where MPRE61 sites coincide with overlaps among CVSI BACs. MPRE61 sites coincide with 3 pairs of overlapping BACs, 1 (AC145413 and AC144901), 2 (AC139879, AC140099) and 3 (AC146932 and AC087424). In addition, an MPRE61 site coincided with a group of 3 overlapping BACs, including AC138784, AC138788 and AC138786. Overlapping BAC pair 2 had two single base differences, and pair 3 had 3 single base differences and one 43 bp insertion/deletion. No sequence differences were observed within MPRE61 for pair 1 or the group of 3 overlapping BACs.

To put the apparent allelic diversity rates into context, we examined the genetic sources of the DNA used to construct the BAC library (RPCI-44). The source of DNA for RPCI-44 was a pooled sample with equal contributions from 4 male crossbred pigs each comprised of 3/8 Landrace, 3/8 Yorkshire and ¼ Meishan [28]. The probability of identifying SNP increases with the diversity of genomes sampled. For the cases of 2 overlapping BACs, the probability of sampling different genomes is 87.5%, different breeds is 65.7%, and one BAC of western (Landrace or Yorkshire) origin and the other of Meishan origin is 37.5%. The probability of sampling diverse genomes is higher for the case of 3 overlapping BACs. The probability of sampling more than one genome is 98.4%, more than one breed is 87.9%, and at least one BAC of western origin combined with one BAC of Meishan origin is 56.25%. The fact that we didn't observe SNP in one of the three pairs of overlapping BACs is not that unusual given that the probability of sampling identical genomes with at least one of the 3 pairs of overlapping BACs is 33% (1-.875^3^). On the other hand, the fact that we did not observe SNP within the group of 3 overlapping BACs given the relatively high probabilities of diverse genomes being sampled is unexpected.

**Figure 4:**
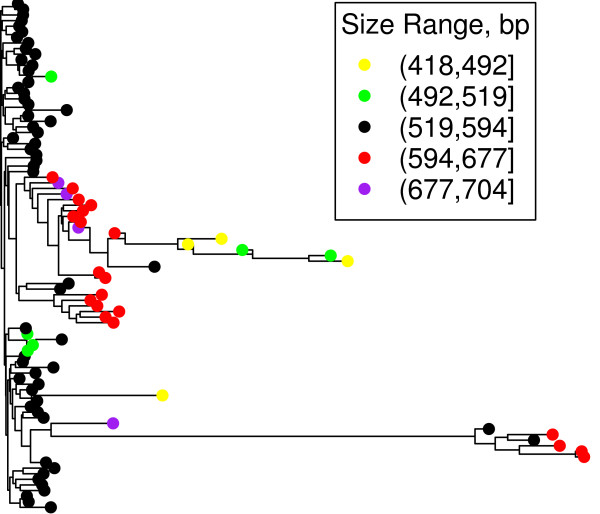
**Diversity of MPRE61 across ten breeds of pig**. This phylogram displays the variety of sequences obtained by amplifying MPRE61 in 16 DNA samples from ten breeds of pig. Size differences are highlighted using colored dots according to the legend. Size cut-offs were chosen to lie between modes of the size distribution which were well separated.

To bolster the relatively small number of distinct MPRE61 loci (7) identified in the CVSI BACs, we further investigated the prevalence and diversity of MPRE61 by cloning and sequencing PCR amplification products derived from 16 pigs sampled from 10 breeds (Berkshire, Chester White, Duroc, Hampshire, Landrace, Meishan, Pietrain, Poland China, Spot, and Yorkshire). We used primers designed to match the highly conserved parts of MPRE61 to amplify and clone (see Methods for details) multiple and variable loci for the RE that are differentiable by size as well as sequence. The different breeds showed indistinguishable smears on denaturing PAGE gels including many different sizes. Too many fragments and too many sizes were present to identify allelic differences in sizes among animals. The PCR products were sequenced to yield 91 reads that were not bacterial or vector contamination. The 91 sequences (listed as a fasta file in Additional file 4) were analyzed with Clustal X (creating a dendrogram file, Additional file 5) and displayed in Figure 4 as a phylogenetic tree. The topology of the tree (number of diverse nodes) is consistent with the estimated copy number of 300 sites in the whole genome given in Table 1. We speculate that the more similar sequences represented as tips close (with few sequence differences) to their common ancestor are probably allelic differences at the same locus. On the other hand, the more diverse tips and peripheral nodes probably represent different sites or loci. The amount of sequence diversity presented in Figure 4 supports the idea that individual integration sites (loci) and alleles of repetitive elements can be uniquely identified by high-throughput array based assays by hybridizing samples to short probes. This demonstrates that repetitive DNA with similar properties to MPRE61 (*i.e*., prevalence and diversity) can be harnessed for genetic and physical mapping [29]. This dispels the long standing myth that repetitive DNA should always be avoided because it is intractable. Our results indicate that some classes (low to intermediate copy number and highly diverse) of repetitive DNA would be tractable with high-throughput technologies.

MPRE61 size differences are not randomly distributed throughout the phylogenetic tree. Different sizes cluster on different branches of the tree; however, the clustering is not complete. This indicates that insertions and deletions (evolutionary events that cause size differences) occurred throughout the evolution of MPRE61, and in some cases while the element was still replicating. The incomplete clustering of sizes indicates evolutionary plasticity and as a result recurrent insertions and deletions.

**Figure 5:**
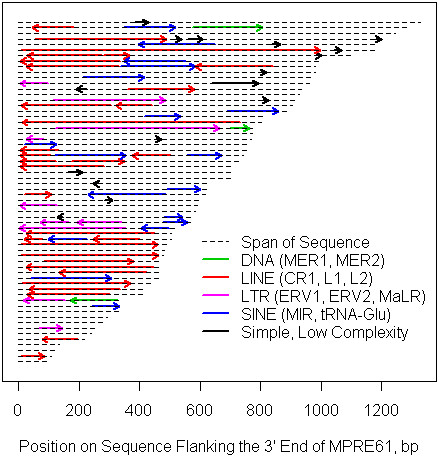
**Repeat elements that flank the 3' end of MPRE61**. The repeat content of 62 BAC-end sequences flanking the 3' end of MPRE61. The origin on the horizontal axis is the last position that matches the 3' end (minimum position within the repeat of 1000 out of the full 1059 bp length) of MPRE61. The 62 flanking sequences are ordered with the longest at the top and the shortest at the bottom. The horizontal position is the distance from the 3' end of the hit to MPRE61. Colored arrows are superimposed on the dotted outline of the flanking sequence to indicate the repeat elements that RepeatMasker found.

MPRE61 was further characterized by plotting BLAST hits of it to the 275,595 sequences in the trace archive of BAC-ends submitted by Sanger. These were plotted along with the repeat elements recognized by RepeatMasker. The most interesting observations included the fact that three times among the 140 hits a PRE1 was incorporated into MPRE61. PRE1 is a porcine specific SINE that is included in the RepeatMasker library. Several other examples existed of PRE1 next to a section of MPRE61, but the trace end occurred next to the PRE1, so that it may or may not have had the continuing section of MPRE61 on its other side. No other REs were found to be incorporated into MPRE61, suggesting that MPRE61 replicated relatively recently. Another interesting observation was that the density of REs on the 3' side of MPRE61 was much higher than on the 5' side. To take a closer look at this, we collected the trace sequence 3' of the 62 hits that ended near (within 60 bp of) the 3' end of MPRE61 (length of 1059 bp). This flanking sequence, ranging in length from 12 to 1368 nucleotides, was analyzed for repeat content and distance of that content from the end of MPRE61 (Figure 5). Running RepeatMasker on the entire collection (275,595 sequences) of Sanger BAC-ends shows that the number of SINE elements is 75% greater than the number of LINE elements (203,206 vs. 116,107). The LINEs tend to be longer than the SINEs, so the total percentage of sequence occupied by the LINEs is actually larger (13.29% vs. 10.29%). The most obvious feature of Figure 5 is that LINEs are significantly over represented on the 3 prime side of MPRE61, particularly in the region closest to the end of MPRE61. For the 22 LINEs that occur within 80 bp of the end of MPRE61, 15 are oriented on the opposite strand and 7 on the same strand. At this point, there is no way to know which strand of MPRE61 might be transcribed. We arbitrarily chose one of the strands and used it consistently. Because the LINEs have a particular internal structure, the 5' and 3' ends are well defined. So another way of looking at the result would be to say that the LINEs occur on the 5' end of MPRE61 (or rather, its reverse complement) with 15 on the same strand and 7 on the opposite strand. Either way, there is less strand conservation than would be expected if MPRE61 used LINEs as a vehicle for either replication or integration.

## Conclusion

From our experience, it seems that although some available programs may help with the process of identification of REs, a level of judiciousness is also required. The BLAST and phylogenetic analyses are proven to be useful to improve the efficacy, particularly when comparisons are made across species. Discovering the RE in one dataset and characterizing their prevalence and diversity in another was crucial to our effort.

Using an approach similar to previously published work but modified to fit our specific goals and data, several repetitive elements were identified in porcine and bovine genomes that do not exist in mouse or human. These elements do not contain signatures of previously identified retrotransposons, but seem to have undergone replication and mutation. Because these elements are in a lower copy number than most of the REs that make up mammalian genomes, they could be exploited in mapping or whole-genome association studies. As the porcine genome sequencing effort progresses, we should know more about the distribution, history and possible contribution of these repeats to the genomic architecture in artiodactyls.

The genuine challenge of genome sequencing and assembly would be enhanced with an improved understanding of repeat elements and their distributions, especially those repeat elements that are species specific.

## Methods

### Bioinformatics

Two hundred-twenty fully sequenced porcine BACs generated by the Comparative Vertebrate Sequencing Initiative [30,31] were downloaded from the RPCI-44 clone library, totaling 36.4 Mb. RepeatMasker [17] masked out 36% of this sequence. All unmasked fragments of sequence that were at least 50 bp long were compared (BLAST) to the original data set. The BLAST parameters used were those recommended by Korf *et al*. (2003) for finding repeat elements, namely -r 1 -q -1 -G 2 -E 2 -W 9 -F "m D" -e 1 for NCBI-BLAST [32]. The output, which contained 1,334,953 hits, was analyzed using two similar methods. One was to use the RECON software [10] downloaded from its website [33] and the other used separate, original PERL scripts that performed several of the same functions included in the RECON package.

### PCR and sequencing

Primer pairs for amplification of genomic DNA were designed from consensus MPRE61 sequences using Primer3 [34]. Primer sequences were 5'-TTTTCCTGTGGTGATTTGTGA-3' and 5'-GGGCGCTGGACTGCTCAAA-3' (positions 278–298 and 953-935 (5' to 3' on opposite strand) of MPRE61, respectively). PCR was performed in a PTC-225 DNA engine (MJ Research Inc, Watertown, MA) using 0.25 U Hot Star^® ^*Taq *polymerase (Qiagen, Valencia, CA, USA), 1X of supplied buffer, 1.5 mM MgCl2, 200 μM dNTPs, 0.8 μM each primer, and 100 ng of genomic DNA in 25 μl reactions. The PCR mixture was held at 94°C for 15 min, and cycled 44 times at 94°C for 20 sec, held at 57°C annealing temperature for 30 sec and extension at 72°C for 1.5 min, followed by a final extension at 72°C for 5 min. Five μl of the PCR reaction was electrophoresed in 1.5% agarose gels to determine quality of amplification and a portion (2–4 μl) was used for cloning in pCR4-TOPO vector (Invitrogen, Carlsbad, CA). Plasmid DNA was prepared using standard alkaline lysis and PTFE filter plates (Millipore, Bedford, MA) and was sequenced with T7 primer.

## Authors' contributions

RW performed the bioinformatic analysis and drafted the manuscript. DN carried out the molecular genetics studies and helped draft the manuscript. JK designed and coordinated the study and helped draft the manuscript. All authors have read and approved the final manuscript.

## Supplementary Material

Additional file 1 **Fasta file of novel porcine repetitive elements**. Each definition line includes an accession number along with the start and end positions for that repetitive element. Click here for file

Additional file 2 **Fasta file that provides the sequences used to create Figure **3. The definition lines include the accession number with the start and end positions of the sequence. Click here for file

Additional file 3 **Dendrogram file used to create Figure **3. The format is the standard output of ClustalX and can be read by various tree viewing and tree making software, including R when using the packages sequinr and ape. Each vertex is labelled consistently with the corresponding fasta file. Click here for file

Additional file 4 **Fasta file of cloned MPRE61 sequences**. The definition line refers to an arbitrary sequence ID generated at USMARC. Click here for file

Additional file 5 **Dendrogram file of cloned MPRE61 sequences used to construct Figure **4. The format is the standard output of ClustalX and can be read by various tree viewing and tree making software, including R when using the packages sequinr and ape. Each vertex is labelled consistently with the corresponding fasta file. Click here for file

## Figures and Tables

**Table 1:** Count numbers for novel porcine repetitive elements

***Repeat name***	***length***	***GC content***	***BLAST hits to SSGI***^***1***^	***BLAST hits to BAC-ends***^***2***^	***count number regular***^***3***^	***count number irregular***^***4***^	***BLAST hits to Bovine Genome***^***5***^
MPRE2	111	0.40	66	528	513		1000
MPRE3	411	0.51	25	392		324	57
MPRE6	255	0.55	15	392		342	0
MPRE11	76	0.33	888	8876	8040		1599
MPRE12	199	0.47	26	272		123	1051
MPRE14	234	0.57	29	292	306		1157
MPRE15	912	0.50	56	520	592	452	1475
MPRE16	276	0.46	379	4688	5051	4035	1260
MPRE17	870	0.29	5	89		75	1002
MPRE19	125	0.34	30	577	550		534
MPRE21	595	0.48	16	189		201	1604
MPRE22	166	0.46	6	83	81		0
MPRE26	324	0.50	75	475	479		1054
MPRE28	140	0.64	27	700	648		0
MPRE38	176	0.35	610	7567	7110	5806	1417
MPRE42	220	0.39	160	2350	2425		1050
MPRE44	55	0.40	22	560	551		6
MPRE49	221	0.50	3	52		50	6
MPRE50	136	0.35	40	907	871		0
MPRE51	71	0.49	17	140	112		110
MPRE52	341	0.30	28	457	703	362	643
MPRE54	326	0.46	17	121	125		62
MPRE55	161	0.52	39	247	244		1075
MPRE58	196	0.41	4	98		98	1034
MPRE59	123	0.27	207	1830	1723		1431
MPRE60	151	0.40	13	98	90		0
MPRE61	1059	0.37	2	31		41	10

^1 ^The number of BLAST hits, at least half as long as the repeat element, found within the TIGR *Sus scrofa *Gene Index version 11, which contains 104,328 entries and 70.0 MB. ^2 ^The number of similar BLAST hits to the Sanger archive of BAC-ends that has 275,595 entries totaling 335.9 MB. ^3,4 ^The regular and irregular columns give the number of BLAST hits across the repeat element, again using the Sanger data. The regular values are the average of the middle 90% of the repeat element while the irregular values are the minimum value within the middle 80% of the repeat element. ^5 ^The number of BLAST hits, including those less than half the length of the repeat element, found within the whole Bovine genome (build AAFCO2).
